# Understanding DNA interactions in crowded environments with a coarse-grained model

**DOI:** 10.1093/nar/gkaa854

**Published:** 2020-10-12

**Authors:** Fan Hong, John S Schreck, Petr Šulc

**Affiliations:** School of Molecular Sciences and Center for Molecular Design and Biomimetics at the Biodesign Institute, Arizona State University, Tempe, AZ 85287, USA; School of Molecular Sciences and Center for Molecular Design and Biomimetics at the Biodesign Institute, Arizona State University, Tempe, AZ 85287, USA; Department of Chemistry, Drexel University, Philadelphia, PA 19104, USA; School of Molecular Sciences and Center for Molecular Design and Biomimetics at the Biodesign Institute, Arizona State University, Tempe, AZ 85287, USA; Center for Biological Physics, Arizona State University, Tempe, AZ 85287, USA

## Abstract

Nucleic acid interactions under crowded environments are of great importance for biological processes and nanotechnology. However, the kinetics and thermodynamics of nucleic acid interactions in a crowded environment remain poorly understood. We use a coarse-grained model of DNA to study the kinetics and thermodynamics of DNA duplex and hairpin formation in crowded environments. We find that crowders can increase the melting temperature of both an 8-mer DNA duplex and a hairpin with a stem of 6-nt depending on the excluded volume fraction of crowders in solution and the crowder size. The crowding induced stability originates from the entropic effect caused by the crowding particles in the system. Additionally, we study the hybridization kinetics of DNA duplex formation and the formation of hairpin stems, finding that the reaction rate *k*_on_ is increased by the crowding effect, while *k*_off_ is changed only moderately. The increase in *k*_on_ mostly comes from increasing the probability of reaching a transition state with one base pair formed. A DNA strand displacement reaction in a crowded environment is also studied with the model and we find that rate of toehold association is increased, with possible applications to speeding up strand displacement cascades in nucleic acid nanotechnology.

## INTRODUCTION

The interactions between DNA/RNA strands are controlled by orthogonal base pairing of adenine (A) to thymine (T) and cytosine (C) to guanine (G) and are essential for fundamental cellular activities and practical molecular therapeutic and diagnostic purposes, such as gene replication (1), gene regulation (2) and diagnostics (3) as well as anti-sense oligonucleotide drugs (4). Furthermore, recent years have also witnessed the emerging field of DNA nanotechnology, which uses DNA to build complex designed molecular nanostructures and molecular machines by taking advantage of its programmability and predictable interactions (5–8), allowing for unprecedented precise control of structure and dynamic behavior at the nanoscale. The properties of DNA strand interactions, such as the thermodynamics and kinetics of duplex formation, determine the assembly efficiency and stability of the DNA structures that affect cellular functions, anti-sense drug efficiency, and the performance of designed molecular machines (1–4,9). Consequently, a quantitative understanding of the biophysical properties of DNA interactions is required to rationalize fundamental cellular functions, as well as to improve nucleic acid-based biotechnologies.

Biophysical properties of DNA and RNA have been well-studied in diluted aqueous solutions (10,11), and have been successfully applied to DNA (and RNA) secondary structure prediction (10,12,13) and molecular probe design for molecular diagnostics and next-generation sequencing (14,15). However, the interaction parameters extracted from a diluted solution do not necessarily correspond to DNA and RNA interactions *in vivo*. In living cells, the environment is occupied with macro-molecules, cell organelles, and relatively small molecules, such as metabolites and osmolytes, occupying 10–40% of the total volume (16–18), resulting in highly crowded and complicated conditions (19,20). Researchers have found that RNA and DNA have significant differences in the thermodynamic and folding properties between the crowded environments and aqueous solutions (21–24). This macromolecular crowding in a cellular environment has a significant influence on both the intra-molecular and inter-molecular interactions by changing the energetic and transport properties of the molecules in the crowding environment.

To improve the performance of nucleic acid-based technologies and our understanding of DNA/RNA-related cellular activities, comprehensive studies of DNA interactions in the crowded environment are necessary and of great importance. Generally, the kinetic and thermodynamic properties of DNA/RNA are investigated through single-molecule and UV melting experiments under crowded conditions achieved *in vitro* by introducing crowding agents (such as PEG, sucrose, urea, dextran and others (25)) into the solution. The comparison of the results between the aqueous solution and the crowded environment is then used to infer principles governing the influence of the crowders on the biophysical properties.

DNA-DNA interactions are complicated processes involving diffusion, nucleation, and zippering steps (26). Although experimental studies can capture the overall results, which can be used to create a simplified physical model to describe the crowding effects (23,25,27–29), the individual steps of the hybridization or duplex formation cannot be easily captured by the experimental methods. As a complement to the experiments, computational modeling, such as molecular dynamics (MD), can provide more detailed information about the process of DNA hybridization in a crowded environment. Ideally, a fully atomistic MD simulation would be used to simulate the hybridization process, but the time-scales involved in such processes are limited by rare events (such as the creation and breaking of bonds) and make such a study infeasible. To address these challenges, a coarse-grained model is required to reduce the computational complexity while at the same time retaining the key information about the molecule. Prior computational studies have investigated the entropic stabilization of a folded RNA state in a crowded environment (30,31), and the role that crowders play in polymer looping kinetics (20).

**Figure 1.**
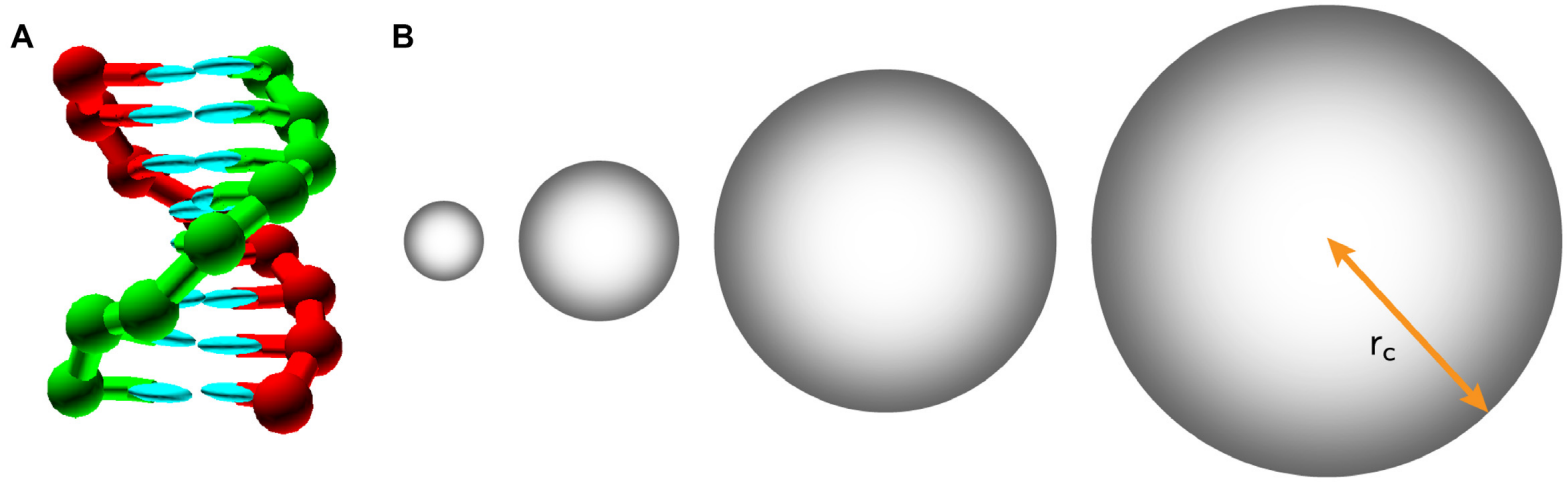
The crowder-oxDNA model. (**A**) A DNA 8-mer duplex configuration, as represented in the oxDNA model. (**B**) The crowder particles with different radii *r*_c_ corresponding to the crowder sizes studied in the simulations, shown in relative size comparison with the DNA 8-mer.

Here we use a coarse-grained DNA model to study the DNA interactions under various crowding conditions. The oxDNA model (32–34) is a coarse-grained DNA model that captures the structural, thermodynamic and mechanical properties of both single-stranded DNA and double-stranded DNA. Where available, the model has been able to quantitatively reproduce experimental measurements of DNA properties and has been successfully applied to the study of duplex and hairpin formation (26,32,35), DNA behavior under a pulling force and torque (36,37), DNA origami assembly (38–40), properties of polyhedral and tile-based nanostructures (41–43), and other complex processes such as strand displacement kinetics (44) and DNA walkers (9,45). We extend the oxDNA2 model (34) by introducing inert crowding particles, which are represented by spheres interacting with excluded volume (schematically shown in Figure 1). In this work, we focus purely on the entropic effect of the excluded volume interaction on the thermodynamics and kinetics of the interactions, by neglecting other interactions between the crowders and DNA, such as electrostatic attraction or repulsion.

With the extended coarse-grained model, we first study the thermodynamics of a DNA 8-mer duplex and a hairpin with a stem of 6-nt and different loop lengths. We find that the crowders increase the melting temperature of both the DNA duplex and the hairpin. Increasing the volume fraction stabilizes the compact state (formed duplex or hairpin) with respect to the unbound state. The increased stability originates from the entropy change caused by the crowding particles in the system. For a fixed volume fraction, we observe smaller crowders having a much higher stabilizing effect than larger crowders, and is due to their higher number density. We fit an analytical formula based on scaled particle theory (SPT) (19,46–48) to our thermodynamic data, and find semi-quantitative agreement in the predicted change in the binding free energy. Differences between the extended oxDNA2 model and SPT are observed for smaller crowder sizes and higher volume occupied by crowders, where the approximations made by SPT are no longer applicable.

Additionally, we performed kinetic simulations of duplex and hairpin formation with individual nucleotide resolution. The rate-determining step of the two transitions is the nucleation step which involves the first hydrogen bond forming between the bases. We find that crowding effects generally favor the association rate *k*_on_, while the dissociation rate *k*_off_ is only weakly affected. The increase in *k*_on_ comes mostly from increasing the probability of reaching the transition state with one base pair formed, thus accelerating the association kinetics. Finally, we study a strand displacement reaction (44) under different crowding conditions. We find that the binding to the toehold region is also increased in crowded environments. As a result, crowded environments could potentially be used to enhance strand displacement cascades rates in nanotechnological applications.

## MATERIALS AND METHODS

### Extending the oxDNA model to include spherical crowders

OxDNA is a coarse-grained model that treats a DNA strand as a chain of rigid bodies, with each rigid body representing a single nucleotide. The interaction potentials in the model, schematically illustrated in Supplementary Figure S1, have been parameterized to reproduce basic structural, thermodynamic and mechanical properties of double-stranded and single-stranded DNA (32,34,49). Each nucleotide has one interaction site to represent the backbone and two sites for the base (one for a stacking interaction and one for a hydrogen-bonding interaction). Hydrogen-bonding and stacking interactions drive the formation of duplexes. The model has been shown to accurately represent the properties of duplexes as well as single-stranded DNA (26,32,49,50).

We introduce here the extended crowder-oxDNA model, where we implement a crowding interaction in the latest oxDNA2 model (34). The crowders are modeled to mimic hard spheres, each having a given radius of *r*_*c*_, as seen in Figure 1B. Crowder particles are assumed to interact with each other and with the nucleotides in the DNA strands through an excluded volume interaction only, which is described by a Lennard–Jones potential. The details are provided in the Supporting Information (Section S1 and Supplementary Table S1). The model also treats electrostatic interactions with the Debye–Hückel potential (with effective backbone charges parameterized to reproduce the changes in duplex and hairpin stability as observed experimentally (34)). However, we use in this work a salt concentration of 1 m for all simulations, which effectively screens all backbone charges, as we do not want the calculation of long-range electrostatic interactions to slow down the simulations. Furthermore, our interest is in studying the effects of inert crowders. A comprehensive study of the effects of charged crowders at low salt concentration will be explored in future work.

For all the simulations performed in this work, we use an averaged strength model, which treats all base-pairing interactions with the same strength. Furthermore, to avoid any undesired base-pairing, we only allow hydrogen-bonding interaction between base pairs that are supposed to form in the duplex (or hairpin stems). These constraints are introduced so as not to perturb the duplex or hairpin formation thermodynamics and kinetics by additional effects, such as the formation of alternative stable secondary structures or sequence effects that would affect the kinetics and thermodynamics and would need to be deconvolved from the effects of the crowders. The effects of stable alternative base-pair formation on hybridization kinetics have been explored previously (26,35) with the oxDNA model, as well as the effects of strong (GC-rich) and weak (AT-rich) segments on duplex formation formation (26) and hairpin formation (33,34,50). In most hybridization and strand displacement experiments, sequences are chosen to minimize interactions other than the intended base pairing and to avoid the formation of undesired secondary structures, and averaged oxDNA model has previously been shown to reproduce well kinetics and thermodynamics of such systems (44). We furthermore do not compare to any specific experiment, and hence a particular sequence choice would further reduce the generality of our results.

Before computing thermodynamics and kinetics quantities for duplex and hairpin formation, we wanted to check whether the diffusion of ssDNA in oxDNA in the presence of crowders was still Brownian. To do so, we first measured the mean-square displacement (MSD) of the center-of-mass of ssDNA of length 8 bases in crowded environments (Supplementary Figure S3) (*r*_c_ = 0.85 nm or 2.56 nm) with excluded volume fractions ϕ = 0.1 and 0.3, and observed diffusive behavior, with the MSD scaling linearly with time. The ssDNA diffusion is also not affected by the crowders’ diffusion constant (increasing the crowders’ diffusion constant by a factor 3 does not change the measured diffusion coefficient of ssDNA in the crowded environment). However, the ssDNA’s diffusion coefficient changed by a factor of 1.17 when the crowder mass decreased by a factor of 27. When we increase the excluded volume fraction from 0.1 to 0.3 we observe a decrease in the diffusion coefficient of ssDNA by approximately a factor of 1.2.

### Crowder sizes and volume fractions

In cellular environments, there are many types of molecules with various sizes. Large molecules, such as proteins with high molecular weight, will create macro-molecular crowding, while small molecules will have small-molecule crowding effects. To study the crowding effects across these scenarios, the excluded volume fraction of the crowding particles is selected from ϕ = 0.1–0.4 to mimic a cellular environment (17,18). Additionally, the radius of the crowding particles is selected from 0.85 to 2.56 nm to mimic crowding conditions ranging from the small solutes to relatively large proteins.

### Simulation methods

Thermodynamic results in this work are obtained by using the virtual-move Monte Carlo (VMMC) algorithm (51), which in combination with umbrella sampling (US) allows efficient cluster Monte Carlo (MC) simulation of strongly-bound systems and was used previously to study the thermodynamics of DNA duplex and hairpin formations with oxDNA (32,35,49). The MC algorithms estimate the free-energy difference between two states (such as 0 bonds and 1 bond) as a log-difference of the number of steps the simulation spends in each state. The US algorithm is an advanced technique that makes the sampling more efficient (see Supporting Information for details). For the studies of DNA hybridization and DNA hairpin formation, we consider the strands (or stem) bound if there is at least one base pair present between the complementary native base pairs in the duplex (recall we do not allow for any mismatches). The melting temperature, *T*_m_, is taken to be the temperature at which the yield of duplex (or hairpin) is 50%. For the duplex system, we use its yield value extrapolated to the bulk (34,52).

To study the kinetics of association, we use molecular dynamics (MD) simulations. To obtain good statistics for the association simulations, which are dominated by rare events, we used forward flux sampling (FFS). FFS facilitates sampling of a complex transition path ensemble by splitting a rare event into several intermediate stages that are defined by the following two order parameters: the proximity of complementary bases and the number of base pairs formed. The system is considered to pass through a given interface λ if the distance between bases is smaller than a given cutoff value, or when the number of formed base pairs is larger or equal to a given cutoff value. We first run MD simulations to estimate the flux, Φ, through the first interface λ_0_. Multiple simulations are then launched from states that have successfully crossed the first interface. The probability *P*(λ_1_|λ_0_) to reach interface λ_1_ from λ_0_ is estimated as the number of simulations that were started from a configuration at interface λ_1_ that successfully reached λ_2_ without crossing interface λ_−2_. Analogously, the probabilities are estimated for crossing the subsequent *N* interfaces λ_*i*_. The overall reaction rate constant *k* is estimated as $k = \Phi \prod _{i = 0}^N P(\lambda _{i+1} | \lambda _i)$. The definitions of respective interfaces for the study of duplex hybridization, hairpin formation, and strand displacement in crowded environments, along with a detailed description of the method, are provided in the Supporting Information (Supplementary Tables S2–S4 and Supplementary Figure S2).

All MD simulations were performed at 37°C and 1 m salt concentration. Free-energy plots were obtained from VMMC simulations run at the same temperature and salt concentration as in the MD simulations. Melting temperatures were obtained from VMMC simulations run at temperatures within several degrees of the actual measured melting temperature, which was obtained using a free-energy re-weighting method (32,33).

For a crowder of radius *r*_c_, we set its mass in the MD simulation to *m*_*r*_ = *m*_0_(*r*_c_/*r*_0_)^3^, where *m*_0_ corresponds to the mass of a single DNA nucleotide in the oxDNA model, and *r*_0_ = 0.85 nm is the radius of the smallest crowder sphere considered. Hence, we keep the density of the spheres representing the crowders constant and adjust their mass appropriately. We further set the diffusion constant of each crowder of radius *r*_c_ to *D*_r_ = *D*_0_*r*_0_/*r*_c_, where *D*_0_ = 0.24 nm^2^ ps^−1^ is the diffusion coefficient of a single DNA nucleotide in the simulations. We set *D*_0_ to a value that corresponds to about 100 times greater than what was experimentally measured for a 14-mer DNA duplex in water. We intentionally choose the value of *D*_0_ to increase the diffusivity of the particles in the simulations and speed-up the sampling of the transitions (see Supporting Information for details).

## RESULTS AND DISCUSSION

**Figure 2.**
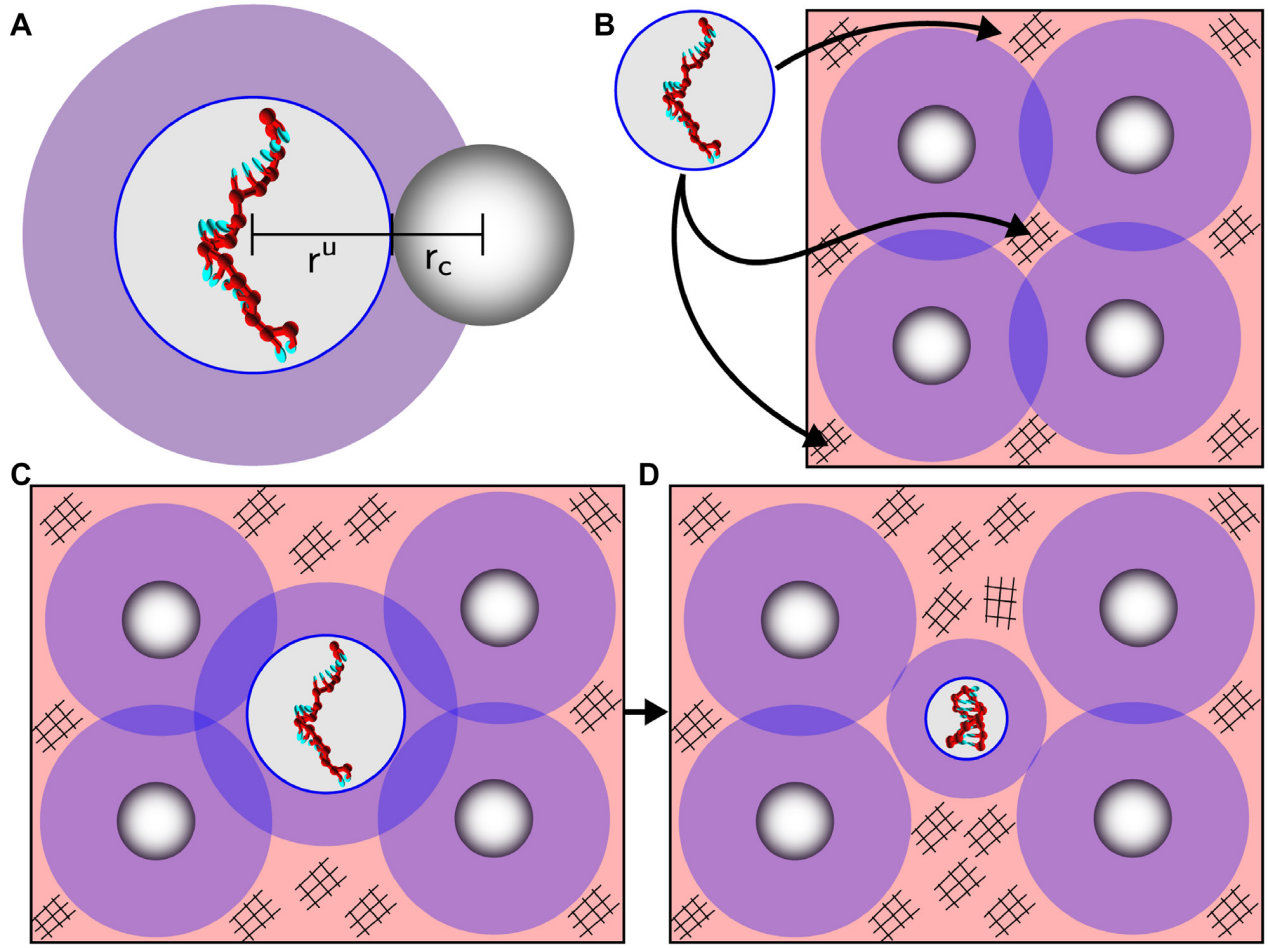
Excluded volume effects of crowders. (**A**) We approximate the disordered single-stranded DNA as a hard-sphere of radius *r*^*u*^. The center-of-mass of the crowder is excluded from the (purple) shaded region of length *r*_c_. Similarly, the center-of-mass of the DNA is excluded from a region (not shown) of length *r*^*u*^ around the crowder. (**B**) The DNA is placed into an environment consisting of spherical crowders with radius *r*_c_, which shows the shaded regions of length *r*^*u*^ excluding the center-of-mass of the sphere representing the DNA strand. Hence, the DNA strand may only be placed into hashed regions and not shaded regions, as it would clash with one of the crowders. Therefore, when the open state of the DNA strand (**C**) folds up into a hairpin (**D**) having radius *r*^*f*^ < *r*^*u*^, more hashed regions become available to the crowders and proteins in the solution (less volume is excluded), and the entropy of the solution goes up, thus making the system with the folded DNA state more free-energetically favorable. An analogous argument can be made for two complementary DNA strands forming a duplex.

We first study the effects of crowders on duplex and hairpin thermodynamics, followed by the kinetics. The entropic effects of crowding on association thermodynamics can be rationalized through scaled particle theory (48), which is intuitively explained in Figure 2. However, the scaled particle argument greatly simplifies the system by modeling the DNA strand as a rigid sphere. We hence first run molecular simulations to estimate the free-energy difference between the bound (base-paired) and unbound states of the system, using a physically accurate representation of DNA strands, the oxDNA model and compare it to the predictions of scaled particle theory. The reasoning for the entropic stabilization of the folded state, however, does not provide information about the kinetics or provide insight into how the association and dissociation rates are affected by the presence of the crowders. For the latter, we need to perform MD simulation and extract the rates, which are discussed further below.

### Crowding effects on DNA duplex and hairpin thermodynamics

We first study the crowding effects on the thermodynamic stability of an 8-mer DNA duplex and hairpins with a 6-nt stem and a 10-nt loop. The former reaction is inter-molecular while the latter is intra-molecular, covering the two most basic nucleic acid interaction types. We used VMMC simulations with oxDNA to obtain the melting temperature and free-energy profiles for both of these systems. The melting temperature is defined as the temperature in which the yield of an open state (for the hairpin), or a dissociated state in the bulk at a given concentration (for the duplex), is 50%. An increase in the melting temperature after introducing crowders would hence correspond to increased stabilization of the duplex (or hairpin) state. The relative stability can also be characterized in terms of the free-energy difference between bound and unbound states, Δ*G*°, which is computed as the free-energy difference between configurations with zero base pairs formed and those with at least 1 base pair formed, e.g. Δ*G°* = *G*_≥1 bp_ − *G*_0 bp_. The relative probability for a system to be in a bound state rather than unbound at temperature *T* is then exp (−Δ*G°*/*k*_B_*T*). It is hence of interest to see how this quantity changes for a system with and without crowders.

**Figure 3.**
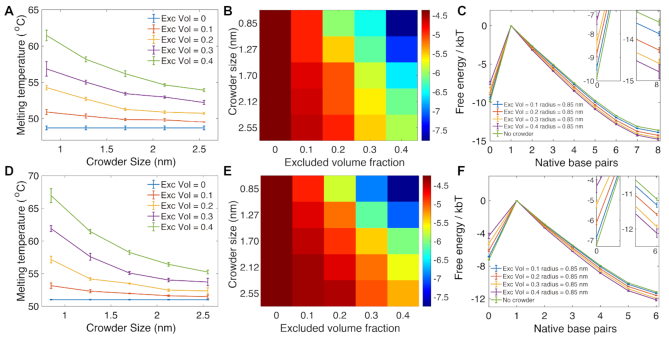
The thermodynamics of DNA duplex formation and hairpin closing in crowded environments. (A, D) The melting temperature of the 8-mer duplex (**A**) and hairpin (**D**) as a function of crowder radius *r*_*c*_ under different crowding volume fractions ϕ. (B, E): The free-energy difference between the unbound and the fully-formed duplex (**B**) and the fully-formed hairpin stem (**E**). (C, F): The free-energy profile of duplex formation (**C**) and hairpin stem formation (**F**) at 37°C. The insets show the profiles around 0 and 6 or 8 bonds. In (A) and (D), the crowder-free case (blue) is shown alongside finite values of ϕ and *r*_c_.

In the simulations, we considered different excluded volume fractions ϕ = 0.1, 0.2, 0.3 and 0.4. For each ϕ, we varied the crowder particle radius *r*_c_ from 0.85 to 2.56 nm with a step-size of 0.42 nm. The change in melting temperatures and free-energy profiles for the duplex and the hairpin as a function of number of base pairs formed are shown in Figure 3. For the crowders with radius *r*_c_ = 0.43 nm, we only consider the volume fraction 0.1 (shown in Supplementary Figure S4A).

Figure 3A and D shows that the crowders enhance the stability of both duplex formation and hairpin closing, respectively, by increasing the melting temperature relative to the crowder-free environments. When the excluded volume remains constant, the melting temperature will decrease as the crowder radius increases. If the size of the crowders remains the same, the larger the number of crowding particles present in the simulation box, the higher the melting temperature, as the stability of the duplex or the hairpin is increased. The melting temperatures for the duplex and the hairpin without crowding particles are 48.7 ± 0.3 and 51.1 ± 0.1°C, respectively. Under the largest crowder volume fraction and smallest crowder radius that we studied (ϕ = 0.4, *r* = 0.85), the melting temperatures increased to 61.5 ± 0.7 and 67.0 ± 1.0°C, for the duplex and hairpin systems, respectively. Similar trends of the crowders’ effect are also seen in the free-energy changes of the duplex and hairpin formation. The heat maps in Figure 3B and E indicate the free-energy difference between the single strand (0 bonds formed) and fully paired states (8 bonds for duplex and 6 for hairpin stem) under different ϕ and *r*_c_ for duplex and hairpin, respectively. For a fixed ϕ, the free-energy difference increases, favoring the fully formed duplex and hairpin, with smaller *r*_c_.

We further plot the free-energy landscapes versus the number of native base pairs formed for the 8-mer and for the hairpin in Figure 3C and F, respectively, for *r*_c_ = 0.85 nm. In the plots, the free energy is set to zero when one base-pair is formed. The profiles for other studied combinations of ϕ and *r*_c_ are shown in the Supporting Information (Supplementary Figures S4 and S5). The landscapes all show that when the excluded volume fraction increases, the single-stranded state with zero base pairs formed becomes less favorable, and at the same time the fully formed duplex (or hairpin stem) state is more stabilized (i.e. has lower free energy).

Supplementary Figures S4 and S5 also show the free-energy dependence on crowder radii *r*_c_ for the duplex and hairpin systems, respectively, varying from 0.85 nm to 2.56 nm at a fixed ϕ. We observe that the differences in the profiles between different *r*_c_ are larger with increasing excluded volume fraction. For a fixed volume fraction, the free-energy difference between states with zero base pairs formed and states with one base pair formed is smaller for smaller *r*_c_. Furthermore, the free-energy difference between a fully-formed duplex (8 bp) or a hairpin stem (6 bp) and the single base pair state is larger for smaller *r*_c_.

This behavior is in accordance with a theoretical analysis based on scaled particle theory (SPT) (48), where the number density of the crowders enhances depletion effects through an increase in the effective osmotic pressure acting on the associating molecules. For fixed excluded volume fraction, the number density will be larger for smaller crowders, and thus their stabilizing effect is more pronounced.

**Figure 4.**
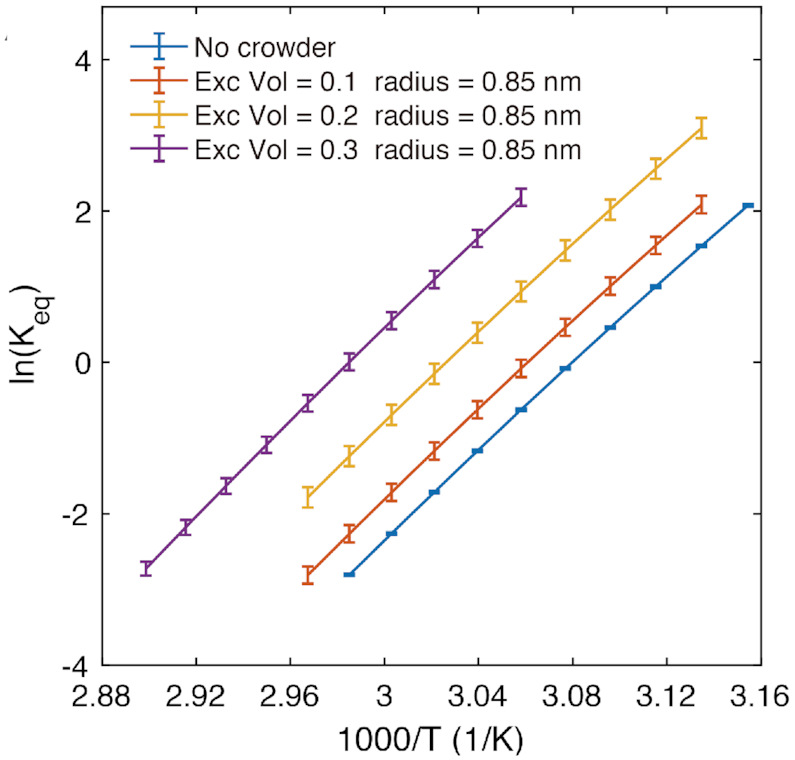
The Van’t Hoff plots for the 8-mer duplex. The entropic contribution is compared for a fixed *r*_c_ = 0.85 nm and ϕ ranging from 0.1 to 0.3.

The effects of the entropic stabilization can be also visualized by plotting the logarithm of the equilibrium constant (ln *K*_eq_) for the duplex (and hairpin) formation as a function of temperature for different crowding conditions. The plots (Figure 4 and Supplementary Figure S6), show the expected linear behavior, with a slope corresponding to the enthalpic difference between bound and unbound states −Δ*H*/*k*_B_, and the intercept corresponding with the entropy change Δ*S*/*k*_B_. The plots show the line shifting to higher entropy as the excluded volume fraction increases for fixed *r*_c_, and shifting to lower entropy with an increase in *r*_c_ for fixed excluded volume fractions, in accordance with our observation from changes in melting temperatures and free-energy profiles.

### Comparing oxDNA thermodynamics with scaled particle theory

**Figure 5.**
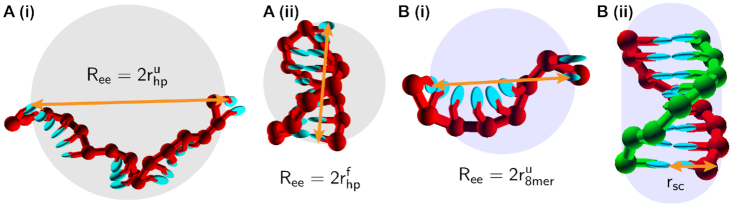
(**A**) The unfolded (i) and folded (ii) states of the hairpin system are illustrated, both showing the end-to-end separation *R*_ee_ (orange arrow) and the radii of the hard sphere used to approximate the DNA in SPT. (**B**) The unfolded (i) and folded (ii) states of the 8-mer duplex system are shown. In (B)(i), only one of the complementary strands is shown with *R*_ee_ (orange arrow) and the hard-sphere radii, while both strands are shown in the formed duplex in (ii) with *r*_*sc*_ illustrating the spherocylinder radius (orange arrow). In all images, spheres or the spherocylinder are not drawn to scale relative to the radii measured for the DNA. The superscripts ‘u’ and ‘f’ in the shown radii refer to ‘unfolded’ and ‘folded’ states, respectively.

Scaled particle theory was developed to approximate the changes to the thermodynamics of polymer folding and assembly when crowded molecules are also present in solution at volume fraction ϕ (19,46,53). It has previously been applied to both protein and RNA folding systems (28,54,55). Here, we apply this theory to DNA hairpin and duplex formation. In SPT the solution of DNA and crowder molecules is assumed to be ideal, and the ssDNA and the crowders are modeled as hard spheres having effective radii $r^u_{\rm hp}$ and *r*_c_, respectively (28), as is illustrated in Figure 5 A(i) for the single strand. The folded hairpin is also modeled as a hard-sphere with radius $r^f_{\rm hp}$ as is shown in Figure 5 A(ii). Figure 5B(i) shows the 8-mer single strand in the duplex system with radius $r^u_{\rm 8mer}$, while in Figure 5 B(ii) the duplex is modeled as a hard spherocylinder with radius *r*_sc_ comprised of two adjacent, non-overlapping spheres that represent the two strands bound together. The radii of the unfolded single-strands are computed in oxDNA by measuring the end-to-end distance *R*_ee_ between terminal bases. See the Supporting Information for more details of the derivation. We compare the SPT models with our simulation results, as the coarse-grained crowder-oxDNA model provides a more detailed and physically accurate representation of double-stranded and single-stranded DNA, but is too complex to extract analytically the predicted free-energy changes as a function of the crowding environment.

In both duplex and hairpin systems, the quantity ΔΔ*G* is computed with SPT as the difference between the free-energies Δ*G°* in crowded and crowder-free environments, where Δ*G°* is the free-energy difference between bound (hairpin or duplex) and unbound states (opened strand or two single strands). Hence ΔΔ*G* < 0 means that in the crowded environment, the bound state is more stable with respect to the unbound state than it was without crowders.

We model unfolded and a folded hairpin configuration as spheres, as shown in Figure 5A(i) and (ii) respectively. We can obtain ΔΔ*G* analytically using SPT (see the Supporting Information for details) and also extract it from oxDNA simulations. The analytical expression for ΔΔ*G*_hairpin_ is derived as a function of radius of for folded and unfolded configurations ($r_{\rm hp}^f$ and $r_{\rm hp}^u$ as shown in Figure 5), the crowder radii *r*_c_, and the excluded volume fraction ϕ. For the duplex, we model the ssDNAs as spheres and the duplex as a spherocylinder (Figure 5B(i) and (ii)) and ΔΔ*G*_duplex_ can be derived in terms of the sphere and spherocylinder sizes $r^u_{\rm 8-mer}$ and *r*_sc_, *r*_c_ and ϕ (55) (see Supporting Information).

**Figure 6.**
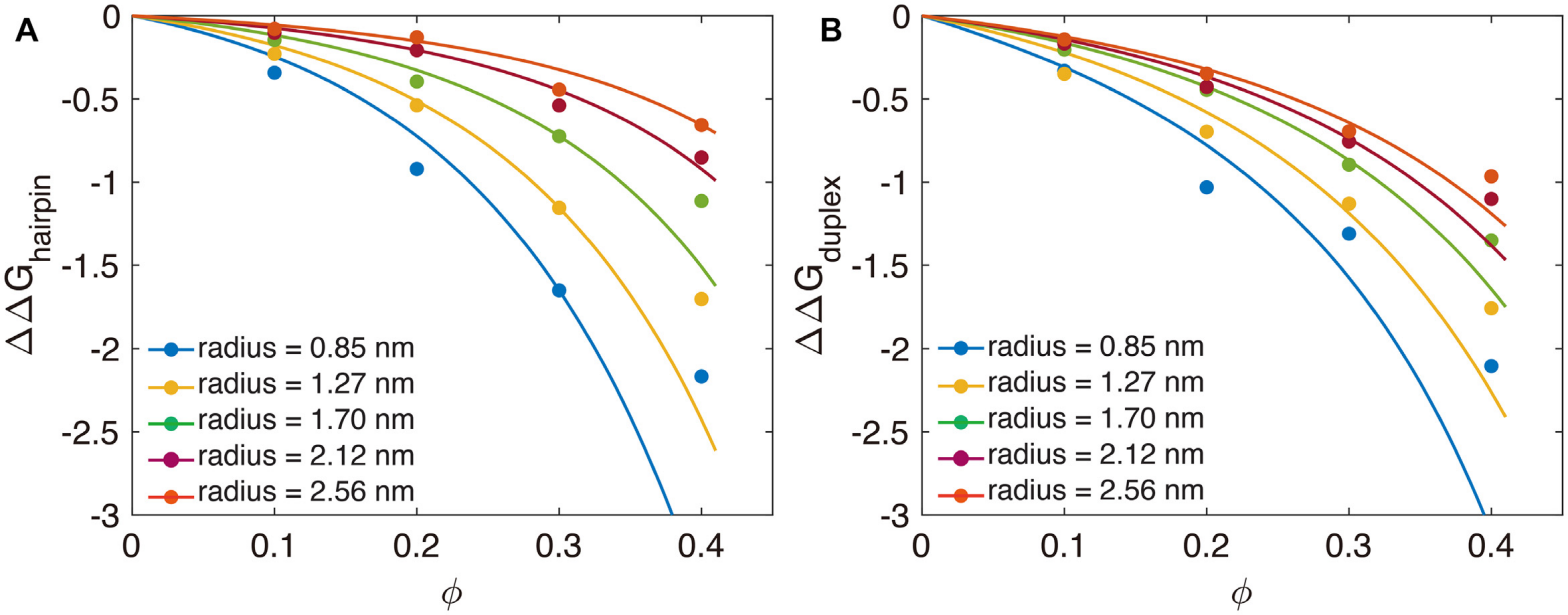
Scaled particle theory predictions (lines) are compared with the relative free energy ΔΔ*G* computed using oxDNA for (**A**) the 4-nucleotide loop hairpin (with 6 bp long stem) and (**B**) the 8-mer duplex (dots).

We use the radii of ssDNA and dsDNA abstracted as spheres and spherocylinders (*r*_sc_, $r^u_{\rm 8mer}$, *r*_hp_) as free parameters to fit the SPT prediction to the measured ΔΔ*G* for hairpin and duplex in oxDNA simulations in environments with and without crowders, respectively (shown as dots in Figure 6). To obtain the fits, Equations S17–S21 and S31–S32 were parameterized using these effective radius parameters, which are listed in Supplementary Table S5.

Figure 6 A and B compare the oxDNA computed ΔΔ*G* and the SPT predictions for the hairpin and the duplex systems with varying crowder sizes and ϕ. Even though our treatment of DNA in SPT is rather simplistic, in both systems, the SPT fit and oxDNA are in near-quantitative agreement for the larger crowder radii (*r*_c_ $\gtrapprox$ 2 nm) and smaller crowder concentrations (ϕ $\lessapprox$ 0.3). However, when *r*_c_ is 2 nm (smaller than the ssDNA), SPT predicts that the crowders influence the thermodynamics of the transition significantly more than the oxDNA model does. Additionally, when the crowder concentration is high, SPT predicts that the crowders influence the free energy of the transition more, as compared to oxDNA for both systems, though these differences are not as large when compared to the small *r*_c_ case. Hence, the SPT provides a good empirical model to predict the entropic effects of the crowders on duplex and hairpin stability but is not accurate enough for cases where the sizes of the crowders are comparable with the size of the individual nucleotide, where the approximation of the entire ssDNA chain as a sphere breaks down.

### Crowding effects on the kinetics of DNA hybridization

**Figure 7.**
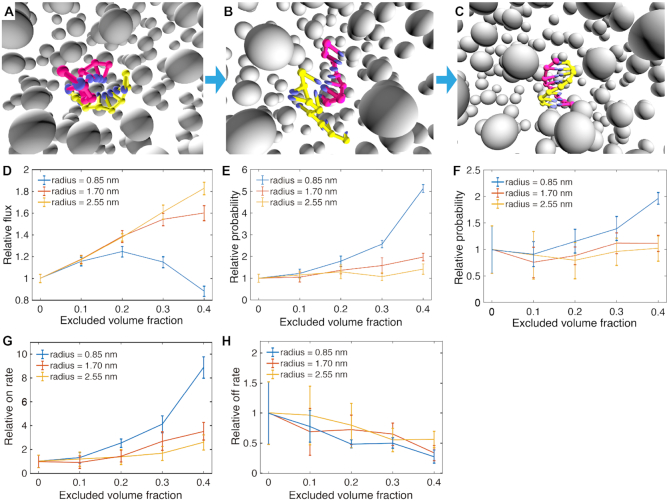
The kinetic FFS study of DNA 8-mer formation in different crowding conditions. (A–C) The illustrations show typical configuration at the three interfaces used to calculate transition rates for the hybridization process: (**A**) proximity between complementary bases, (**B**) the first base pair formed, (**C**) all 8 bp formed. The grey, isolated particles are crowders with the same radius, shown in perspective view. (**D–F**) The relative reaction flux/transition probabilities across the three interfaces, respectively. (**G–H**) The relative *k*_on_ and *k*_off_ for 8-mer duplex formation.

**Figure 8.**
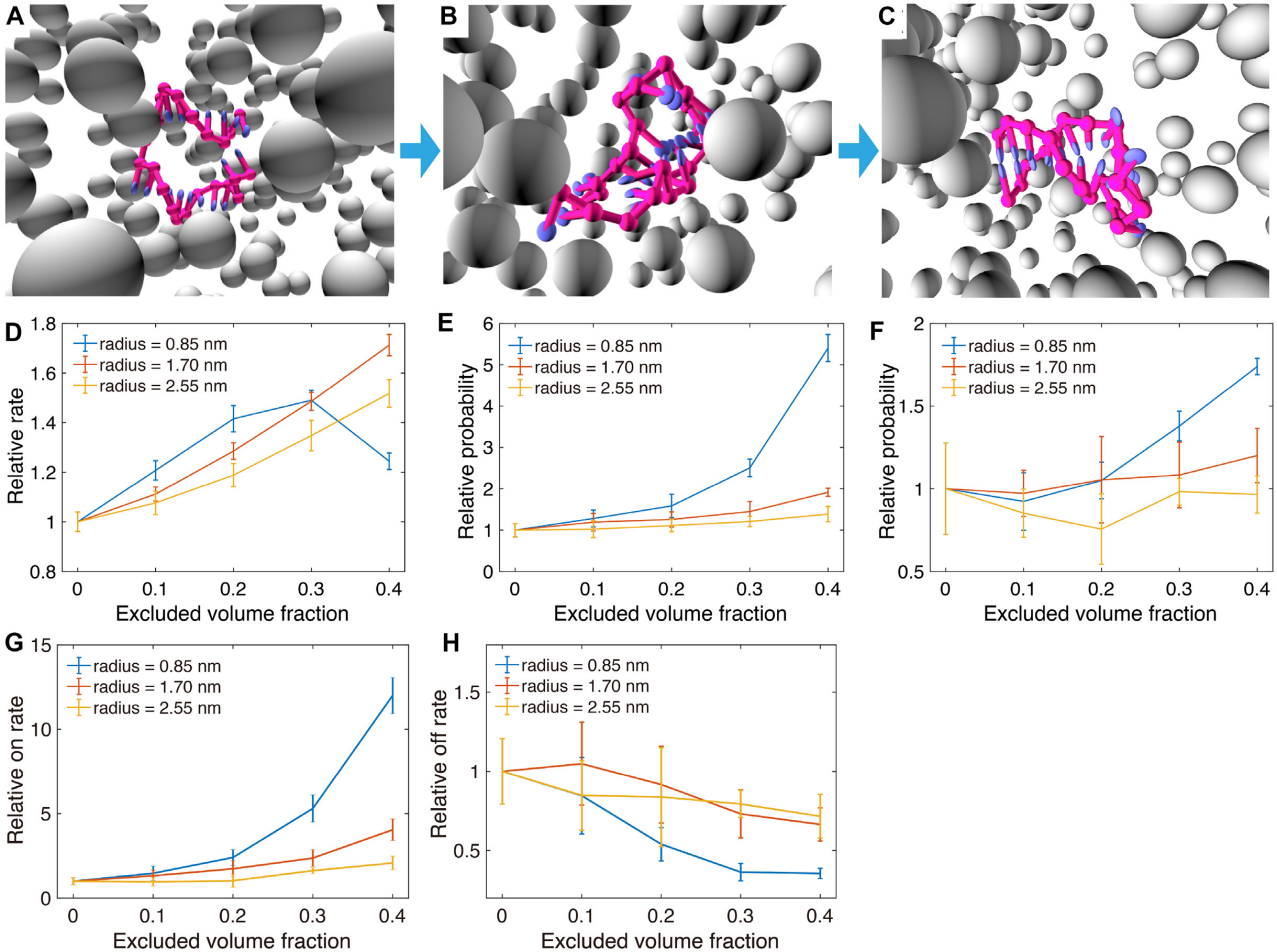
The kinetic study of hairpin closing with the crowder-oxDNA model. (A–C) A typical DNA hairpin formation process represented in three stages: (**A**) the stem bases getting close, (**B**) the formation of first base pair and (**C**) the full base pairing of the stem. (**D–F**) The relative reaction rate of the three stages, respectively. (**G–H**) the overall relative *k*_on_ and *k*_off_.

We next explore how the presence of crowders affects the kinetic behavior of DNA during association using the crowder-oxDNA model. Specifically, we investigate the hybridization kinetics of the DNA 8-mer (Figure 7), the hairpin formation with stem length of 6 and loop length of 10 (Figure 8), and additionally a hairpin formation with a stem length of 6 and a loop length of 4.

We first simulate the duplex hybridization and hairpin stem formation with no crowders present using the oxDNA model. All reported rates, fluxes, and transition probabilities for crowded systems are normalized with respect to the mean values measured with no crowders present. Additionally, all kinetic simulations were carried out at 37°C and with at a salt concentration of 1 m.

We next perform kinetic studies of DNA in crowded environments with *r*_c_ = 0.85, 1.70 and 2.56 nm. For each *r*_c_ considered, we study the kinetics of duplex and hairpin stem formation for ϕ = 0.1, 0.2, 0.3 and 0.4, respectively. Additionally, we only consider the hairpin with loop length 4 at *r*_c_ = 0.85 nm and ϕ = 0.1 and 0.4 (see Supplementary Figure S8). We use FFS, as described in the Materials and Methods section and in the Supporting Information, to sample the kinetics of duplex and stem formation, and obtain flux and transition probabilities.

The hybridization of DNA strands requires the diffusion of two complementary bases to spatial proximity, followed by the creation of a few base pairs to initiate a ‘zippering’ up of the rest unpaired bases (26). The 8-mer hybridization and hairpin closing processes are divided into three stages, schematically shown in Figures 7A–C and 8A–C respectively. First, we calculate the flux through the first interface where the minimum distance between complementary bases is 0.85 nm. The relative normalized fluxes are shown in Figures 7D and 8D, which compare different crowder conditions. We then sample the transition probabilities going from states where the minimum distance between complementary bases is ≤0.85 nm to those where one base pair has formed. The relative probabilities are shown in Figures 7E and 8E for duplex and hairpin, respectively. The final interface, shown in Figures 7F and 8F, measures the transition probability from one base pair formed to a fully-formed duplex or hairpin stem, respectively. The overall *k*_on_, obtained from multiplying the values obtained from the flux and transition probabilities, are shown in Figures 7G and 8G for duplex and hairpin, respectively. For each case studied, we also obtained *k*_off_, which is shown in Figures 7H and 8 for the two systems, by using the relation

(1) $$\begin{equation*} k_{\rm off} = k_{\rm on} e^{\Delta G^0/ k_{\rm B} T}, \end{equation*}$$

where Δ*G°* was obtained as the free-energy difference between bound and unbound states (Figure 3B and E) from VMMC sampling simulations under the same crowding conditions as in the FFS simulations where we measured *k*_on_.

Analysis of the binding kinetics shows similar trends both for the 8-mer duplex and the hairpin. Figure 7D shows the relative fluxes (relative to that measured for the crowder-free case), which measure the rate at which complementary bases come into close spatial proximity. The plot shows that the flux increases linearly as a function of the excluded volume occupied by crowders with *r*_c_ = 2.56 nm. For *r*_c_ =  1.70 nm, the increase in flux is similar but becomes slightly slower when the excluded volume fraction ϕ reaches 0.3. However, for *r*_c_ = 0.85 nm, the flux shows non-monotonic behavior with increasing excluded volume fraction, first increasing until reaching a plateau at ϕ = 0.2, then decreasing thereafter. This phenomenon is likely due to competition between caging effects, which favor states at proximity, and increased viscosity caused by the crowders (20). The confinement effect will help the DNA strands make contact more quickly since crowders fill up space in the system, and hence reduce the search space for complementary bases. At the same time, crowders also increase the viscosity and become obstructors that can hinder contact between complementary pairs. Similar effects are observed for the hairpin with loop length 10, where the flux for the smallest crowder (0.85 nm) decreases only after the volume fraction reaches 0.3 (Figure8 D). However, for the hairpin with loop length 4, the flux through the first interface decreases with increasing crowder volume fraction (Supplementary Figure S8), indicating that the obstruction effect dominates for hairpins with shorter loop lengths. We note, however, that the overall differences in flux between different volume fractions and crowder sizes are all within a factor 2 for all cases studied.

After complementary bases encounter each other over a small spatial separation, base-pairing may take place (Figure 7A and B). For this transition, shown in Figure 7E, the relative transition probability increases for all crowder sizes considered with increasing crowder volume fraction. Since two DNA molecules are already spatially close to each other, the viscosity effect is likely not as important as the confinement effect on the nucleation of the hybridization.

At 40% volume fraction, the relative transition probabilities are 5.1 ± 0.2, 2.0 ± 0.2 and 1.4 ± 0.2 for the three studied crowder radii, respectively. The reaction rate boost by the crowders mainly comes from the increased probability to create the first base pair. This supports the previously stated hypothesis (28) that excluded volume effects lead to an increase in *k*_on_ by stabilizing the binding transition state.

Finally, in the last ‘zippering’ step shown in Figure 7F, the crowders have a slightly positive effect on the transition probability to the fully formed duplex, especially for the smaller crowders. The improvement may still come from the improved stability by the confinement effects.

The overall reaction on-rates, which are the product of the flux and respective transition probabilities of the three stages, are shown in Figure 7G. For a fixed crowder radius, if we increase the excluded volume fraction ϕ, *k*_on_ will increase monotonically within the range of 0–40%. For a fixed ϕ, *k*_on_ will decrease with an increasing crowder radius. Under the excluded volume of 40%, *k*_on_ for the crowder sizes considered are 8.9 ± 0.9, 3.5 ± 0.7 and 2.6 ± 0.6, respectively.

We also calculated the off-rate *k*_off_ for comparison, which is shown in Figure 7F. We plot the relative *k*_off_, normalized with respect to the value calculated for the system with no crowders. The relative off rates for different *r*_c_ are shown in Figure 7H. The relative off rate decreases with increasing excluded volume fraction, but the crowding effects have smaller effects on *k*_off_ than on *k*_on_.

We further studied the effects of the mass of the crowder and the crowder diffusion coefficient on the association kinetics of the 8-mer, for crowders with radii *r*_c_ =  1.70 nm and excluded volume fraction ϕ = 0.2. As is shown in Supplementary Figure S7, we found that increasing the mass of crowders by a factor of 3, or increasing of diffusion coefficient by a factor of 3, has negligible effects on the measured association rate *k*_on_, and within error-bars, the mean *k*_on_ remains the same for all masses and diffusion coefficients considered.

The kinetics of hairpin formation is also thoroughly studied with the model, using the same FFS protocol as in the case of duplex formation. For the hairpin system, we used the following interfaces for the flux and transition probability calculations: (i) complementary bases in the native stem region encounter each other over a small spatial range (Figure 8A); (ii) the creation of one base pair in the stem region (Figure 8B) and (iii) the fully-formed stem, which is when 6 base pairs have formed (Figure 8C).

Overall, the crowder has a similar effect on hairpin closing that it had on duplex formation. The relative reaction rate *k*_on_ increases monotonously with an increasing excluded volume fraction. Under the highest excluded volume we simulated (40%), the relative reaction rates are 12.0 ± 1.1, 4.0 ± 0.6 and 2.1 ± 0.4 for the crowder radii 0.85, 1.70 and 2.55 nm, respectively. Hence, the confinement effect has a slightly stronger influence on the intra-molecular interactions (stem formation) compared to what was observed for inter-molecular duplex formation.

To understand how crowders influence hairpin folding when the loop length is varied, we also performed a kinetic study on a hairpin with a loop length of four nucleotides with *r*_c_ = 0.85 nm under different excluded volume fractions (0–40%), using the same FFS protocol that we employed for hairpin of loop length 10 (data are shown in Supplementary Figure S8). For the relative flux, the hairpin with a loop length of 4-nt shows a monotonous decrease with increasing excluded volume fraction. The probability of forming the first base pair from spatially proximal complementary bases in the stem is similar for excluded volume fractions 0.1 and 0.3. However, at ϕ = 0.4, the hairpin with loop length 10-nt has a higher relative reaction rate to form one base pair, while for the last interface (which measures the probability of stem formation given 1 formed base pair), the shorter loop has a slightly higher success probability than the 10-base loop. Overall, for the crowder radius *r*_c_ = 0.85 nm, the *k*_on_ rate for hairpin formation is similar for loop length 4 and loop length 10 at ϕ = 0.1 and ϕ = 0.2. However, *k*_on_ of stem formation with loop length 4 is smaller for larger excluded volume fractions (3.6 ± 0.2 for ϕ = 0.3 and 6.5 ± 0.4 for ϕ = 0.4) than *k*_on_ for the longer loop under the same crowding conditions. The results suggest that the crowders have stronger viscosity effects on the hairpin with the shorter loop length, as the first two stages of formation (reaching end-to-end proximity and forming the first base pair) are the same or lower for the short loop in all excluded volume fractions investigated. The short-loop hairpin also likely benefits less from the caging effects of the crowders.

We further calculated Δ*G°* for the hairpin with loop length 4 (Supplementary Figure S9) in combination with Eq. (1) to obtain the relative *k*_off_ at *r*_c_ = 0.85 nm. The quantity decreases by up to a factor of 2.5 at $40\%$ crowding fraction, similar to values obtained with the hairpin with loop length 10. Hence, the main difference in crowding effects between loop length 10 and 4 results from a change in *k*_on_.

### Strand displacement under crowded environments

**Figure 9.**
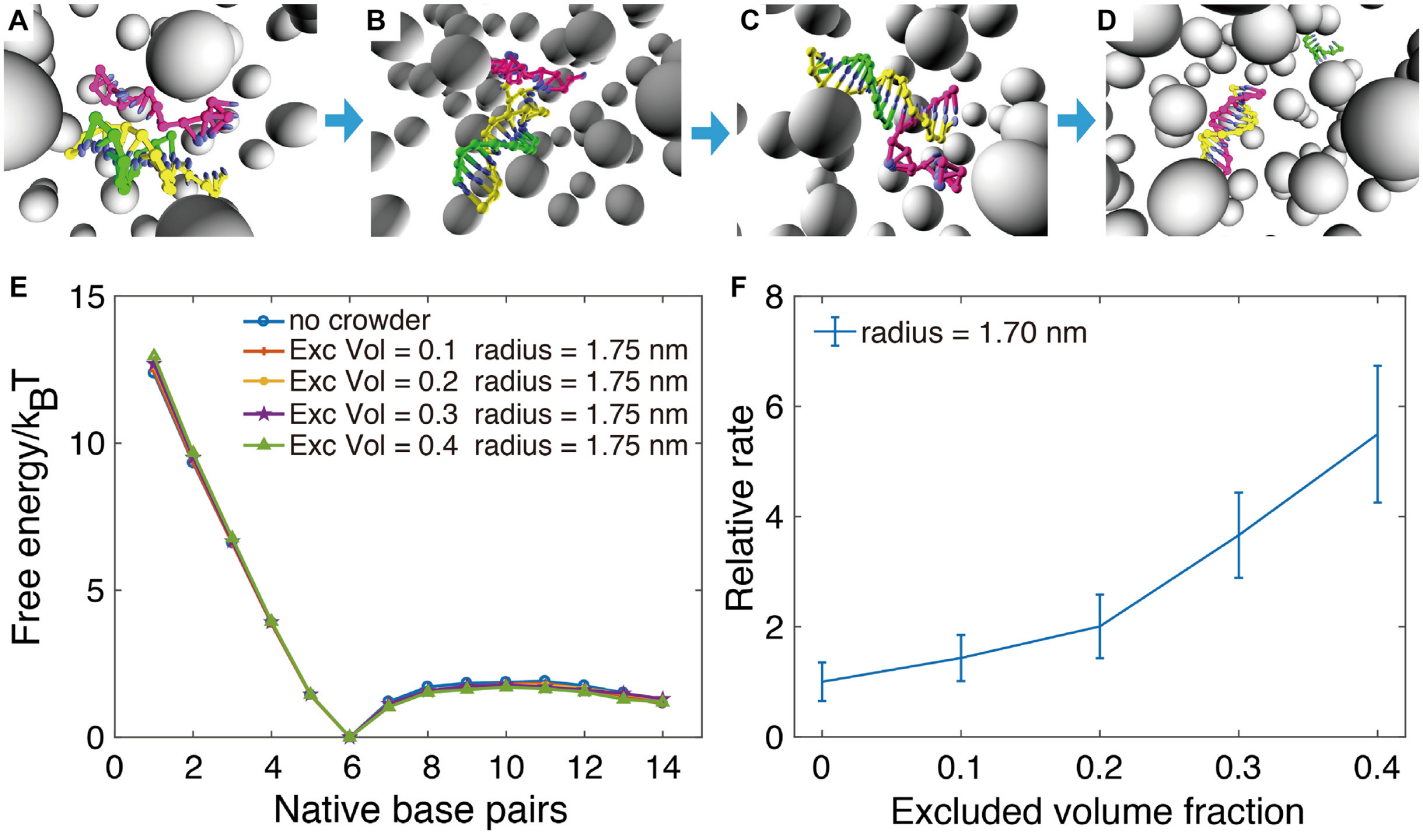
DNA strand displacement reaction in a crowded environment. (**A–D**) A snapshot of a typical configuration of the system crossing different interfaces: the contact of the toehold region, the first bond formation in the toehold region, the toehold region fully paired, and finally the full displacement of the incumbent strand. (**E**) The free-energy profile versus inter-strand base pair formation between the invader strand and the substrate strand. (**F**) The overall reaction rate of strand displacement under different excluded volume fraction with crowder radius of 1.70 nm

Nucleic acid strand displacement is a reaction that allows one (invader) single-stranded DNA to displace another (incumbent) strand that is paired to a third (substrate) strand, as is illustrated in Figure 9A–D. The invader strand (shown in red) will displace the incumbent strand (green) from the substrate strand (yellow) through a 6-nt toehold and then a branch migration process for 10-nt. Strand displacement processes are of great importance in active nucleic acid nanotechnology (6,56) and are likely involved in several processes in biology (57). To study how crowders affect the strand displacement reaction, we conducted both VMMC and FFS simulations for the strand displacement process to extract the thermodynamic and kinetic features of the reaction. In these simulations, the crowder radius was taken to be  1.70 nm at excluded volume fractions up to $40\%$.

The free-energy profile of base-pair formation between the invader strand and the substrate strand is shown in Figure 9E, starting from the initial toehold association to branch migration, and finally to the fourteenth base pair formed. Generally, the free energy will first decrease after the first base pair forms as more stabilizing base pair contacts are created in the toehold association process, and then overcome a free-energy barrier to initiate the branch migration (44). We also compared the free-energy profile in the toehold binding step with that of the 8-mer duplex hybridization, from 1 base pair to 6 base pairs having formed, and found it to be similar to the toehold binding profile (shown in Supplementary Figure S10). During the branch migration process, after crossing the initial barrier, the free-energy profile is mostly flat and does not show any significant change between the reaction under different excluded volumes.

To study how the crowders affect the kinetics of strand displacement, FFS is used to study the process by dividing it into four stages: (i) the contact of the toehold region, (ii) the first base pair forming in the toehold region, (iii) full base pairing in the toehold region and (iv) the branch migration process, as shown in Figure 9A–D. The relative rate of the strand displacement as a function of excluded volume fraction is shown in Figure 9F. The relative rate monotonously increases up to about 5.5 ± 1.2 for ϕ = 0.4. The relative flux and transition probabilities for the individual interfaces are shown in Supplementary Figure S11, which show that the contribution to the overall augmentation in *k*_on_ with increasing ϕ comes from an increase in the binding rate of the toehold, the same as what was observed for the duplex formation kinetics with *r*_c_ =  1.70 nm. The presence of crowders has almost no effect on the branch migration kinetics (Supplementary Figure S11D) of the strand displacement reaction.

The overall study of the strand displacement in crowded environments suggests a possible strategy to increase the interaction rate of strand displacement cascades in DNA nanotechnology, by including crowders in the solution to speed-up the binding rate to the toehold region, thus accelerating the reaction.

## CONCLUSION

In summary, to investigate DNA duplex hybridization and hairpin closing under crowded conditions, we have extended the previously developed coarse-grained oxDNA model by adding crowder interactions. The thermodynamic study revealed that crowders can stabilize DNA duplex and hairpin formation through purely an entropic origin. The kinetics simulations show that most of the change in free energy comes from speeding-up the binding rate, while the unbinding rate only changes slightly. These thermodynamic and kinetic trends are also consistent with prior experimental studies of RNA (28), as well as with coarse-grained simulations for the folding of the WW peptide domain (58,59). By dissecting the duplex and hairpin formation into a series of stages, we found that the reaction rate boost mainly comes from the stabilization of the intermediate transition state with one base pair having formed. However, we also note opposing effects, where the speed of association of complementary regions is partially hindered by collisions with crowders. Overall the stabilization of the transition state is the dominant effect and the association rate always increases with increasing occupied volume fraction. For a constant volume fraction, the effects of crowder sizes have also been explored, and we found that the thermal stabilization (and corresponding speed-up in *k*_on_) is larger for smaller sizes of crowders, in agreement with scaled particle theory (SPT) predictions.

Furthermore, we have provided a detailed comparison of the thermodynamics of hairpin and duplex formation with effective SPT that abstracts DNA strands and hairpins as spheres and duplexes as spherocylinders. We find that in the regime of large crowders and small excluded volume fraction, SPT can fit simulation thermodynamics data with effective radius parameters, but the simplified spherical description assumed by SPT cannot reproduce behavior for excluded volume fraction over 30% and crowder sizes below  1.70 nm because it is no longer accurate enough to capture the entropic effects of the crowders on the DNA thermodynamics.

Nucleic acid interactions in cellular environments are complicated processes since the crowding particles have various shapes, sizes, and electrostatic properties. The coarse-grained model developed here can be further extended to include more interactions to mimic more complex systems, such as long-range electrostatic interactions, linear chains of particles that mimic polymeric crowders such as PEG, and be used to obtain insight into nucleic acid interactions in complex cellular environments. The present model, however, presents an important reference for comparison with other modeling and experimental efforts, as it captures the thermodynamic and kinetic effects solely based on excluded volume interactions with the crowders. Hence, any deviations from the simulation results have to originate from effects and processes not included in the current model, such as long-range electrostatic interactions or crowder-crowder and crowder-DNA binding affinities. Even though our analysis has been performed with a coarse-grained model of DNA, we expect the obtained results for duplex hybridization, hairpin formation, and strand-displacement would also apply to RNA molecules.

Finally, as the role that crowders play in DNA processes is not yet well characterized by experiments, we suggest that future endeavors consider the polymeric crowders PEG, dextran, and ficoll. The PEG 8000 crowder was used by Dupuis *et al.* who reported good agreement between experiment and scaled-particle theory for the changes to the thermodynamics of a DNA hairpin when crowders were added to the solution (28). Their results suggest that the spherical modeling of the polymer chain was not too approximate for volume fractions under 20%, and they showed that steric interactions between the DNA and the crowder were the cause for the observed change in hairpin stability. Additionally, the synthetic polymers dextran-70 and ficoll have also been used extensively in the protein aggregation literature as molecular crowding agents (although some studies have pointed to enthalpic interactions between protein and crowder).

## DATA AVAILABILITY

The source code for the crowder-oxDNA model and the scripts and files for the setup of simulations carried out in this work are available on github.com/sulcgroup/crowderoxdna.

## Supplementary Material

gkaa854_Supplemental_File

## Notes

Present address: Fan Hong, Wyss Institute for Biologically Inspired Engineering, Harvard University, Boston, MA 02115, USA.

Present address: John S. Schreck, National Center for Atmospheric Research, Boulder, CO 80305, USA.

## References

[1] Akabayov B., Akabayov S.R., Lee S.-J., Wagner G., Richardson C.C. Impact of macromolecular crowding on DNA replication. Nat. Commun. 2013; 4:1615. 23511479 10.1038/ncomms2620 PMC3666333

[2] Rosi N.L., Giljohann D.A., Thaxton C.S., Lytton-Jean A.K., Han M.S., Mirkin C.A. Oligonucleotide-modified gold nanoparticles for intracellular gene regulation. Science. 2006; 312:1027–1030. 16709779 10.1126/science.1125559

[3] Khodakov D., Wang C., Zhang D.Y. Diagnostics based on nucleic acid sequence variant profiling: PCR, hybridization, and NGS approaches. Adv. Drug Deliv. Rev. 2016; 105:3–19. 27089811 10.1016/j.addr.2016.04.005

[4] Askari F.K., McDonnell W.M. Antisense-oligonucleotide therapy. N. Engl. J. Med. 1996; 334:316–318. 8532029 10.1056/NEJM199602013340508

[5] Hong F., Zhang F., Liu Y., Yan H. DNA origami: scaffolds for creating higher order structures. Chem. Rev. 2017; 117:12584–12640. 28605177 10.1021/acs.chemrev.6b00825

[6] Zhang D.Y., Seelig G. Dynamic DNA nanotechnology using strand-displacement reactions. Nat. Chem. 2011; 3:103. 21258382 10.1038/nchem.957

[7] Pinheiro A.V., Han D., Shih W.M., Yan H. Challenges and opportunities for structural DNA nanotechnology. Nat. Nanotechnol. 2011; 6:763. 22056726 10.1038/nnano.2011.187 PMC3334823

[8] Seeman N.C., Sleiman H.F. DNA nanotechnology. Nat. Rev. Mater. 2018; 3:17068.

[9] Khara D.C., Schreck J.S., Tomov T.E., Berger Y., Ouldridge T.E., Doye J.P., Nir E. DNA bipedal motor walking dynamics: an experimental and theoretical study of the dependency on step size. Nucleic Acids Res. 2018; 46:1553–1561. 29294083 10.1093/nar/gkx1282 PMC5814849

[10] SantaLucia Jr J., Hicks D. The thermodynamics of DNA structural motifs. Annu. Rev. Biophys. Biomol. Struct. 2004; 33:415–440. 15139820 10.1146/annurev.biophys.32.110601.141800

[11] Yin Y., Zhao X.S. Kinetics and dynamics of DNA hybridization. Acc. Chem. Res. 2011; 44:1172–1181. 21718008 10.1021/ar200068j

[12] Mathews D., Sabina J., Zuker M., Turner D. Expanded sequence dependence of thermodynamic parameters improves prediction of RNA secondary structure. J. Mol. Biol. 1999; 288:911–940. 10329189 10.1006/jmbi.1999.2700

[13] Hofacker I.L. Vienna RNA secondary structure server. Nucleic Acids Res. 2003; 31:3429–3431. 12824340 10.1093/nar/gkg599 PMC169005

[14] Tsourkas A., Behlke M.A., Rose S.D., Bao G. Hybridization kinetics and thermodynamics of molecular beacons. Nucleic Acids Res. 2003; 31:1319–1330. 12582252 10.1093/nar/gkg212 PMC150230

[15] Zhang J.X., Fang J.Z., Duan W., Wu L.R., Zhang A.W., Dalchau N., Yordanov B., Petersen R., Phillips A., Zhang D.Y. Predicting DNA hybridization kinetics from sequence. Nat. Chem. 2018; 10:91. 29256499 10.1038/nchem.2877 PMC5739081

[16] Fulton A.B. How crowded is the cytoplasm?. Cell. 1982; 30:345–347. 6754085 10.1016/0092-8674(82)90231-8

[17] Ellis R.J. Macromolecular crowding: obvious but underappreciated. Trends Biochem. Sci. 2001; 26:597–604. 11590012 10.1016/s0968-0004(01)01938-7

[18] Mourao M.A., Hakim J.B., Schnell S. Connecting the dots: the effects of macromolecular crowding on cell physiology. Biophys. J. 2014; 107:2761–2766. 25517143 10.1016/j.bpj.2014.10.051 PMC4269789

[19] Zhou H.-X., Rivas G., Minton A.P. Macromolecular crowding and confinement: biochemical, biophysical, and potential physiological consequences. Annu. Rev. Biophys. 2008; 37:375–397. 18573087 10.1146/annurev.biophys.37.032807.125817 PMC2826134

[20] Shin J., Cherstvy A.G., Metzler R. Kinetics of polymer looping with macromolecular crowding: effects of volume fraction and crowder size. Soft Matt. 2015; 11:472–488. 10.1039/c4sm02007c 25413029

[21] Nakano S.-i., Karimata H., Ohmichi T., Kawakami J., Sugimoto N. The effect of molecular crowding with nucleotide length and cosolute structure on DNA duplex stability. J. Am. Chem. Soc. 2004; 126:14330–14331. 15521733 10.1021/ja0463029

[22] Kilburn D., Roh J.H., Guo L., Briber R.M., Woodson S.A. Molecular crowding stabilizes folded RNA structure by the excluded volume effect. J. Am. Chem. Soc. 2010; 132:8690–8696. 20521820 10.1021/ja101500g PMC2906142

[23] Knowles D., LaCroix A.S., Deines N.F., Shkel I., Record M.T. Separation of preferential interaction and excluded volume effects on DNA duplex and hairpin stability. Proc. Natl. Acad. Sci. U.S.A. 2011; 108:12699–12704. 21742980 10.1073/pnas.1103382108 PMC3150925

[24] Spitale R.C., Flynn R.A., Zhang Q.C., Crisalli P., Lee B., Jung J.-W., Kuchelmeister H.Y., Batista P.J., Torre E.A., Kool E.T. et al. Structural imprints in vivo decode RNA regulatory mechanisms. Nature. 2015; 519:486. 25799993 10.1038/nature14263 PMC4376618

[25] Nakano S.-i., Miyoshi D., Sugimoto N. Effects of molecular crowding on the structures, interactions, and functions of nucleic acids. Chem. Rev. 2013; 114:2733–2758. 24364729 10.1021/cr400113m

[26] Ouldridge T.E., Šulc P., Romano F., Doye J.P., Louis A.A. DNA hybridization kinetics: zippering, internal displacement and sequence dependence. Nucleic Acids Res. 2013; 41:8886–8895. 23935069 10.1093/nar/gkt687 PMC3799446

[27] Kilburn D., Roh J.H., Behrouzi R., Briber R.M., Woodson S.A. Crowders perturb the entropy of RNA energy landscapes to favor folding. J. Am. Chem. Soc. 2013; 135:10055–10063. 23773075 10.1021/ja4030098 PMC3773054

[28] Dupuis N.F., Holmstrom E.D., Nesbitt D.J. Molecular-crowding effects on single-molecule RNA folding/unfolding thermodynamics and kinetics. Proc. Natl. Acad. Sci. U.S.A. 2014; 111:8464–8469. 24850865 10.1073/pnas.1316039111 PMC4060727

[29] Baltierra-Jasso L.E., Morten M.J., Laflör L., Quinn S.D., Magennis S.W. Crowding-induced hybridization of single DNA hairpins. J. Am. Chem. Soc. 2015; 137:16020–16023. 26654490 10.1021/jacs.5b11829

[30] Denesyuk N.A., Thirumalai D. Crowding promotes the switch from hairpin to pseudoknot conformation in human telomerase RNA. J. Am. Chem. Soc. 2011; 133:11858–11861. 21736319 10.1021/ja2035128

[31] Denesyuk N.A., Thirumalai D. Entropic stabilization of the folded states of RNA due to macromolecular crowding. Biophys. Rev. 2013; 5:225–232. 28510164 10.1007/s12551-013-0119-x PMC5425724

[32] Ouldridge T.E., Louis A.A., Doye J.P. Structural, mechanical, and thermodynamic properties of a coarse-grained DNA model. J. Chem. Phys. 2011; 134:02B627. 10.1063/1.3552946 21361556

[33] Šulc P., Romano F., Ouldridge T.E., Rovigatti L., Doye J.P., Louis A.A. Sequence-dependent thermodynamics of a coarse-grained DNA model. J. Chem. Phys. 2012; 137:135101. 23039613 10.1063/1.4754132

[34] Snodin B.E., Randisi F., Mosayebi M., Šulc P., Schreck J.S., Romano F., Ouldridge T.E., Tsukanov R., Nir E., Louis A.A. et al. Introducing improved structural properties and salt dependence into a coarse-grained model of DNA. J. Chem. Phys. 2015; 142:234901. 26093573 10.1063/1.4921957

[35] Schreck J.S., Ouldridge T.E., Romano F., Šulc P., Shaw L.P., Louis A.A., Doye J.P. DNA hairpins destabilize duplexes primarily by promoting melting rather than by inhibiting hybridization. Nucleic Acids Res. 2015; 43:6181–6190. 26056172 10.1093/nar/gkv582 PMC4513862

[36] Matek C., Ouldridge T.E., Doye J.P., Louis A.A. Plectoneme tip bubbles: coupled denaturation and writhing in supercoiled DNA. Sci. Rep. 2015; 5:7655. 25563652 10.1038/srep07655 PMC5224516

[37] Engel M.C., Smith D.M., Jobst M.A., Sajfutdinow M., Liedl T., Romano F., Rovigatti L., Louis A.A., Doye J.P. Force-induced unravelling of DNA origami. ACS Nano. 2018; 12:6734–6747. 29851456 10.1021/acsnano.8b01844

[38] Snodin B.E., Romano F., Rovigatti L., Ouldridge T.E., Louis A.A., Doye J.P. Direct simulation of the self-assembly of a small DNA origami. ACS Nano. 2016; 10:1724–1737. 26766072 10.1021/acsnano.5b05865

[39] Sharma R., Schreck J.S., Romano F., Louis A.A., Doye J.P. Characterizing the motion of jointed DNA nanostructures using a coarse-grained model. ACS Nano. 2017; 11:12426–12435. 29083876 10.1021/acsnano.7b06470

[40] Snodin B.E., Schreck J.S., Romano F., Louis A.A., Doye J.P. Coarse-grained modelling of the structural properties of DNA origami. Nucleic Acids Res. 2019; 47:1585–1597. 30605514 10.1093/nar/gky1304 PMC6379721

[41] Schreck J.S., Romano F., Zimmer M.H., Louis A.A., Doye J.P. Characterizing DNA star-tile-based nanostructures using a coarse-grained model. ACS Nano. 2016; 10:4236–4247. 27010928 10.1021/acsnano.5b07664

[42] Kočar V., Schreck J.S., Čeru S., Gradišar H., Bašić N., Pisanski T., Doye J.P., Jerala R. Design principles for rapid folding of knotted DNA nanostructures. Nat. Commun. 2016; 7:10803. 26887681 10.1038/ncomms10803 PMC4759626

[43] Reinhardt A., Schreck J.S., Romano F., Doye J. P.K. Self-assembly of two-dimensional binary quasicrystals: A possible route to a DNA quasicrystal. J. Phys. Condens. Matter. 2016; 29:014006. 27830657 10.1088/0953-8984/29/1/014006

[44] Srinivas N., Ouldridge T.E., Šulc P., Schaeffer J.M., Yurke B., Louis A.A., Doye J.P., Winfree E. On the biophysics and kinetics of toehold-mediated DNA strand displacement. Nucleic Acids Res. 2013; 41:10641–10658. 24019238 10.1093/nar/gkt801 PMC3905871

[45] Šulc P., Ouldridge T.E., Romano F., Doye J.P., Louis A.A. Simulating a burnt-bridges DNA motor with a coarse-grained DNA model. Natural Comput. 2014; 13:535–547.

[46] Lebowitz J.L., Rowlinson J.S. Thermodynamic properties of mixtures of hard spheres. J. Chem. Phys. 1964; 41:133–138.

[47] Minton A.P. The influence of macromolecular crowding and macromolecular confinement on biochemical reactions in physiological media. J. Biol. Chem. 2001; 276:10577–10580. 11279227 10.1074/jbc.R100005200

[48] Ping G., Yang G., Yuan J.-M. Depletion force from macromolecular crowding enhances mechanical stability of protein molecules. Polymer. 2006; 47:2564–2570.

[49] Šulc P., Romano F., Ouldridge T.E., Rovigatti L., Doye J.P.K., Louis A.A. Sequence-dependent thermodynamics of a coarse-grained DNA model. J. Chem. Phys. 2012; 137:5101. 10.1063/1.4754132 23039613

[50] Mosayebi M., Romano F., Ouldridge T.E., Louis A.A., Doye J.P. The role of loop stacking in the dynamics of DNA hairpin formation. J. Phys. Chem. B. 2014; 118:14326–14335. 25404188 10.1021/jp510061f

[51] Whitelam S., Geissler P.L. Avoiding unphysical kinetic traps in Monte Carlo simulations of strongly attractive particles. J. Chem. Phys. 2007; 127:154101. 17949126 10.1063/1.2790421

[52] Ouldridge T.E., Louis A.A., Doye J.P. Extracting bulk properties of self-assembling systems from small simulations. J. Phys. Condens. Matter. 2010; 22:104102. 21389436 10.1088/0953-8984/22/10/104102

[53] Reiss H., Frisch H., Lebowitz J. Statistical mechanics of rigid spheres. J. Chem. Phys. 1959; 31:369–380.

[54] Minton A.P. Implications of macromolecular crowding for protein assembly. Curr. Opin. Struc. Biol. 2000; 10:34–39. 10.1016/s0959-440x(99)00045-7 10679465

[55] Minton A.P. The effect of time-dependent macromolecular crowding on the kinetics of protein aggregation: a simple model for the onset of age-related neurodegenerative disease. Front. Phys. 2014; 2:48.

[56] Simmel F.C., Yurke B., Singh H.R. Principles and applications of nucleic acid strand displacement reactions. Chem. Rev. 2019; 119:6326–6369. 30714375 10.1021/acs.chemrev.8b00580

[57] Hong F., Šulc P. An emergent understanding of strand displacement in RNA biology. J. Struct. Bio. 2019; 207:241–249. 31220588 10.1016/j.jsb.2019.06.005

[58] Cheung M.S., Klimov D., Thirumalai D. Molecular crowding enhances native state stability and refolding rates of globular proteins. Proc. Natl. Acad. Sci. U.S.A. 2005; 102:4753–4758. 15781864 10.1073/pnas.0409630102 PMC555696

[59] Cheung M.S., Thirumalai D. Effects of crowding and confinement on the structures of the transition state ensemble in proteins. J. Phys. Chem. B. 2007; 111:8250–8257. 17585794 10.1021/jp068201y

[60] Poppleton E., Bohlin J., Matthies M., Sharma S., Zhang F., Šulc P. Design, optimization and analysis of large DNA and RNA nanostructures through interactive visualization, editing and molecular simulation. Nucleic Acids Res. 2020; 48:e72. 32449920 10.1093/nar/gkaa417 PMC7337935

